# The New Urology Match: How Recent Innovations Including Virtual Interviews and Preference Signaling Have Changed Match Outcomes

**DOI:** 10.7759/cureus.53167

**Published:** 2024-01-29

**Authors:** John Heard, Rushil Y Rawal, Bradley Amazan, Karl-Ray Jeune, Andrew Freedman

**Affiliations:** 1 Urology, Cedars-Sinai Medical Center, Los Angeles, USA; 2 Urology, State University of New York (SUNY) Downstate College of Medicine, New York, USA

**Keywords:** trends, graduate, medical, education, urology, internship and residency

## Abstract

Objectives: To determine how recent changes in the urology match occurring from 2021 to 2023, including virtual interviews (VIs) and preference signals (PS), affected match outcomes.

Methods: The American Urological Association (AUA) match data from 2021 to 2023 was compared to the 15 years prior. This was obtained from the AUA website and a previous study of public AUA match data. Self-reported applicant characteristics and outcomes from the Urology Residency Applicant Spreadsheet 2021-2023 were compared to the four years prior.

Results: Between 2021 and 2023, residency programs offered 43 interviews each, compared to an average of 35 in the 15 years prior. Programs have been receiving more applications each year, from a low of 225 in 2019 to a peak of 347 in 2022. This resulted in an interview offer rate of 13% between 2021 and 2023, compared to 16% in the five years prior. Applicants applied to a mean of 88 programs in 2023, increasing each year since 40 in 2006. Applicants attended 12 interviews on average between 2021 and 2023, compared to 13 in the two years prior. Self-reported applicant data similarly demonstrated that, compared to the four years prior, applicants between 2021 and 2023 applied to more programs (81 vs. 70), had a lower interview offer rate (22% vs. 32%), and a higher interview acceptance rate (90% vs. 75%).

Conclusions: During the years with VIs, programs offered more interviews and applicants attended fewer on average, indicating a larger applicant pool was interviewed. Despite the introduction of PS, applicants applied to more programs in 2022 and 2023 than ever before.

## Introduction

In response to the COVID-19 pandemic, the Society for Academic Urologists (SAU) introduced a number of changes to the 2020-2021 urology residency match. These included recommended limitations on in-person sub-internships, a shift to online programming with virtual interviews (VIs), and a single release date for interview offers, in departure from the previous rolling interview process [1]. While many aspects of medical education transitioned back to in-person in subsequent years, VIs have remained and are likely to continue into the future [2].

Another consequential change to the match process was the introduction of preference signaling (PS) in 2022. This system was created to allow applicants to demonstrate genuine interest in residency programs prior to interview invitations. A significant driver of this change has been the increasing number of applications received each year by residency programs. As program directors (PDs) work to evaluate more applications in a limited amount of time, it becomes more difficult to evaluate applicants holistically, improve resident diversity, and gauge genuine program interest. Initial satisfaction with PS was positive overall, and the program will expand from 5 signals per applicant in the 2022 and 2023 matches to 30 signals in 2024 [3-6].

While the SAU considers future changes to the match process, it is important to consider how these innovations have altered match outcomes. With the completion of the 2023 urology match, there are now three years of data from which to learn about these effects. In this study, we compiled data from the American Urological Association (AUA) and self-reported applicant data from the Urology Residency Applicant Spreadsheet to compare the 2021-2023 match cycles to previous years to better understand the effects of these recent changes on match outcomes.

## Materials and methods

Summary match statistics from the AUA for 2017-2023 were publicly accessible without password protection [7]. Data spanning 2006-2016 was derived from a prior study on public AUA match data [8]. Anonymous urology residency applicant data from 2017-2023 was sourced from the openly accessible Google spreadsheet titled 'Urology Residency Applicant Spreadsheet'. The data from 2017 and 2018 were pre-aggregated on the spreadsheet and are herein referred to as 2017+2018. Applicants lacking a numerical entry for the position matching the rank list were presumed unmatched and subsequently excluded. Virtual sub-internships were omitted from the analysis, and the study received no external funding. Institutional review board approval was unnecessary due to the data's public availability.

For data analysis, Microsoft Excel (Microsoft Corp., Redmond, WA, USA) and SPSS Statistics version 27 (IBM Corp., Armonk, NY, USA) were employed. Continuous variables underwent normal distribution testing via the Shapiro-Wilk test, followed by analysis through Student’s t-tests or Mann-Whitney U tests. Pearson chi-square analysis was applied to assess categorical variables. The predetermined threshold for statistical significance was set at p = 0.05.

## Results

The AUA data

**Figure 1:**
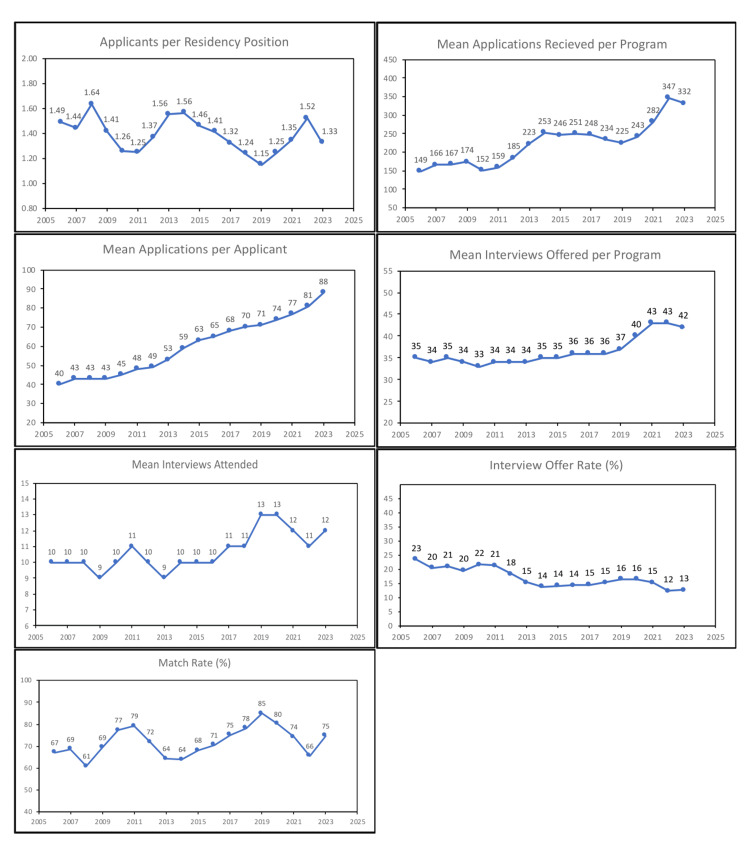
Selected AUA data on application and interview rates by year from 2006 to 2023 AUA: American Urological Association

Publicly available match statistics from the AUA were compared for match years with VIs, i.e., 2021-2023 to the fifteen years prior. The percentage of applicants who are US senior medical students ranged from 70% in 2019 to 92% in 2014 and 2020 (Table 1). The number of applicants submitting rank lists in proportion to residency positions increased from 1.15 in 2019 to 1.52 in 2022 and decreased to 1.33 in 2023 (Figure 1). The previous high was 1.64 in 2008. Applicants have been applying to more programs each year, with 40 in 2006 and 88 in 2023. During the period of VIs in 2021-2023, applicants attended fewer interviews than the two years prior (mean 12 vs. 13), but still more than the historic average (11).

**Table 1:** The AUA match data on applicants by year from 2006 to 2023 AUA: American Urological Association, IMG: International medical graduate

Parameters	2006	2007	2008	2009	2010	2011	2012	2013	2014	2015	2016	2017	2018	2019	2020	2021	2022	2023
Applicants submitting rank list	350	348	404	373	337	339	381	434	446	433	417	422	402	389	441	481	556	508
US senior (%)	78	80	79	87	80	87	87	90	92	77	85	85	85	70	92	77	84	78
US nonsenior (%)	10	10	9	3	7	2	3	2	3	14	8	8	7	20	2	16	11	15
IMG (%)	13	10	12	11	13	11	10	8	6	9	7	8	5	10	6	7	5	6
Ranked applicants per position (n)	1.49	1.44	1.64	1.41	1.26	1.25	1.37	1.56	1.56	1.46	1.41	1.32	1.24	1.15	1.25	1.35	1.52	1.33
Mean programs applied (n)	40	43	43	43	45	48	49	53	59	63	65	68	70	71	74	77	81	88
Mean interviews taken per applicant (n)	10	10	10	9	10	11	10	9	10	10	10	11	11	13	13	12	11	12
Match rate (%)	67	69	61	69	77	79	72	64	64	68	71	75	78	85	80	74	66	75

Residency programs have been receiving more applications each year since the previous low of 225 in 2019 to a peak of 347 in 2022. Forty-three interviews were offered per program in the era of VIs, compared to an average of 35 in the 15 years prior. Despite offering more interviews, the interview offer rate decreased from 16% in 2017-2020 to 13% in 2021-2023, with a notable decrease from 15% to 12% in the 2022 match. Programs received a record 8.1 applications per interview offered in 2022 (Table 2). The match rate for 2021-2023 was 71.3%, compared to 71.8% in the 15 years prior. The match rate of 66% in 2022 was the lowest since 64% in 2014 and higher than the nadir of 61% in 2008.

**Table 2:** The AUA match data on residency programs by year from 2006 to 2023 AUA: American Urological Association

Parameters	2006	2007	2008	2009	2010	2011	2012	2013	2014	2015	2016	2017	2018	2019	2020	2021	2022	2023
Programs (n)	109	110	110	110	114	115	116	117	118	123	124	130	133	136	142	143	143	145
Residency positions (n)	235	241	247	264	268	271	278	279	285	296	295	319	325	339	354	357	365	386
Mean applications received per program (n)	149	166	167	174	152	159	185	223	253	246	251	248	234	225	243	282	347	332
Mean interviews offered per program (n)	35	34	35	34	33	34	34	34	35	35	36	36	36	37	40	43	43	42
Mean interviews offered per position (n)	16.2	15.5	15.6	14.2	14.0	14.4	14.2	14.3	14.5	14.5	15.1	14.7	14.7	14.8	16.0	17.2	16.8	15.9
Mean applications per interview (n)	4.3	4.9	4.8	5.1	4.6	4.7	5.4	6.6	7.2	7.0	7.0	6.9	6.5	6.1	6.1	6.6	8.1	7.9
Program interview rate (%)	23	20	21	20	22	21	18	15	14	14	14	15	15	16	16	15	12	13

Self-reported data

Anonymous self-reported match data was available for 22% (470/2,106) of matched US senior applicants from 2017 to 2023 (Table 3). Applicant characteristics such as United States Medical Licensing Examination (USMLE) scores, Alpha Omega Alpha (AOA) membership, and research productivity fluctuated but were not significantly different during the period with VIs. Applicants applied to significantly more residency programs between 2021 and 2023 (81 vs. 70, p < 0.005); however, this has been increasing each year since 2020. The median number of interviews offered to each applicant (22) had been steady from 2017 to 2020 but was significantly lower during years with VIs at 19 per applicant (p < 0.005). However, that figure has been decreasing each year since 2019. Between 2021 and 2023, applicants received more interview offers from waitlists or cancellations (2 vs. 1, p < 0.01). The interview offer rate, which had been relatively stable, dropped from 32% between 2017 and 2020 to 22% in 2021-2023 (p < 0.00001). The rate at which applicants accepted offered interviews was 90% in 2021-2023, compared to 75% before (p < 0.00001).

**Table 3:** Self-reported US senior applicant match data by year from 2017 to 2023 *N = Number of applicants who self-reported match data

Parameters	2017+2018	2019	2020	2021	2022	2023
N*	137	64	45	73	85	66
Percentage of applicants reporting (%)	24 (137/575)	26 (64/245)	13 (45/337)	25 (73/298)	26 (85/328)	28 (66/323)
Median sub-internships attended (n)	3	3	3	2	2	3
Median # of programs applied (n)	69	74	67	77	79	88
Median interviews offered (n)	22	23	21	19	18	17
Median interviews from waitlist or cancellation (n)	1	2	1	2	1	2
Median interviews attended (n)	15	15	15	16	16	17
Median interview offer rate (%)	31	32	32	27	24	20
Median interview acceptance rate (%)	78	73	70	86	93	92
Median programs on rank list (n)	16	15	16	16	16	16
Median matched position (n)	2	2	2	2	2	2

## Discussion

In this study, we analyzed publicly available match data from the AUA and Urology Residency Applicant Spreadsheet to measure changes in match outcomes after the introduction of VIs and PS. Data from the AUA suggests that while residency programs offered more interviews from 2021 to 2023, each applicant attended fewer interviews, indicating that interviews were distributed to more applicants. This was supported by self-reported data from applicants who applied to more programs but received fewer interview offers between 2021 and 2023.

The increased number of applicants receiving interviews in 2021-2023 resulted in the highest number of applicants interviewed per residency position since 2015. With the overall number of applicants increasing as well, the period with VIs has seen the largest pool of applicants for PDs to choose from in the years for which data is available. Certainly, interviewing more applicants per residency position increases competition and lowers the match rate, but a larger applicant pool also has the benefit of increased access.

Since the transition to VIs, there has been a considerable effort to evaluate their effectiveness, benefits, and costs. In a survey taken after the 2021 match by Spencer et al., a majority of surveyed applicants felt they were able to adequately assess the program and faculty; however, assessments of residents and physical location were more challenging [9]. In another survey, faculty reported that VIs made assessments of applicants' fit, communication skills, and commitment to urology more challenging [10]. These shortcomings were countered by significant benefits in time savings, cost reduction for applicants and programs, and reduced carbon emissions [10-13]. Overall, in 2021, the majority of applicants and program directors (PDs) reported satisfaction with match outcomes and did not believe in-person interviews would have changed them [14]. Surveys taken after the 2022 match indicate that the perception of VIs continued to improve, with more applicants reporting that faculty interviews, resident interviews, and resident socials were well replicated virtually compared to 2021 [3].

Discussion of VIs has also focused on their potential to increase access for underrepresented minorities (URM) and females. While the proportion of URM trainees in urology remained unchanged between 2011 and 2020, the introduction of VIs offered the possibility of improved access for URM applicants, largely through the reduced cost of interviewing, since educational debt is greater and more common for URM students [15,16]. As previously mentioned, application costs decreased significantly due to VIs, and in Movassaghi et al.’s survey after the 2022 match, two-thirds of PDs believed VIs increased access for applicants to attend interviews [17]. Since the transition to VIs, the proportion of female trainees has continued to increase; however, data on the percentage of URM trainees during this time is lacking and warrants further investigation [18]. It will be interesting to see if not only the reduced cost of VIs but also the increased number of interviews offered, as reported in this study, improve URM success in the match.

Attention has also been paid to the effect of the virtual match process on the geography of match outcomes, as it was hypothesized that programs may prefer their own students to those evaluated virtually. In a study on the 2021 match, applicants were found to have a higher likelihood of matching to their home program and AUA section than between 2017 and 2019 [19]. A separate study by Wang et al. found that in 2021, smaller residency programs (one to two residents per year) had a higher rate of home students and in-section matches, while larger programs did not [20]. In the 2022 match, however, geographic dispersion returned to the pre-pandemic pattern. These findings are likely connected to the novelty of VIs in 2021 and pandemic-related limitations on sub-interviews, which were relaxed for 2022 and removed for the 2023 match.

The introduction of PS to the match process from 2021 to 2023 was another significant change. This program was initially proposed as a mechanism for applicants to demonstrate genuine interest in a program prior to the evaluation of their application [21]. One goal of PS was to reduce the number of applications submitted by applicants, which had been increasing since 2006 (Figure 1). Although PS was introduced in the 2022 match and continued with modifications in 2023, applicants applied to the most programs ever (88) in 2023. In fact, the increase of seven applications from 2022 to 2023 was the largest increase among match data since 2006. Applicants viewed PS favorably and desired its continuation in future match cycles in a number of survey studies; however, given its limited effect on the number of applications submitted, further modifications of the PS program may be needed [3-5,22].

The limitations of this study include the limited statistical analysis possible on the summary statistics provided by the AUA. As a result, a number of the findings mentioned above should be interpreted through that lens and viewed as interesting trends rather than definitively demonstrated changes. An additional limitation is the nature of the self-reported data from the Urology Residency Application Spreadsheet. While it is impossible to authenticate the details of anonymous postings on a publicly accessible document, comparisons with published match data in urology and other specialties have found self-reported data to be slightly biased towards more competitive applicants (higher exam scores, more research items), which could limit its external validity [23-25]. Additionally, there are numerous other factors in the match process that likely also played a role in the changes described. These include the return of away sub-internships, virtual open houses and sub-internships, a single release date for interviews, and others.

## Conclusions

The goal of the match process is to create the most optimal match outcomes for applicants and residency programs in the most equitable way. Significant changes to the urology match occurred over the 2021-2023 match cycles, including the introduction of VIs and PS. In association with these changes, residency programs offered more interviews, increasing the size and diversity of the applicant pool, as well as improving access and reducing the costs of the application process. These changes help advance the goals of the urology residency match, and further improvements are encouraged.
